# Effect of First-Line Combination Systemic Therapy on Favorable-Risk Clear Cell Renal Cell Carcinoma: A Retrospective Study

**DOI:** 10.3390/biomedicines14010238

**Published:** 2026-01-21

**Authors:** Soon Il Lee, Minsuk Kwon, Sung Hee Lim, Se Hoon Park

**Affiliations:** 1 Division of Hematology-Oncology, Department of Internal Medicine, Dankook University College of Medicine, Cheonan 31116, Republic of Korea; avnrt@hanmail.net; 2 Division of Hematology-Oncology, Department of Medicine, Samsung Medical Center, Sungkyunkwan University School of Medicine, Seoul 06351, Republic of Korea; minsuk.dr.kwon@samsung.com (M.K.); sunghee1022.lim@samsung.com (S.H.L.)

**Keywords:** kidney neoplasm, favorable risk, systemic therapy

## Abstract

**Background/Objectives**: For patients with advanced or metastatic clear cell renal cell carcinoma (RCC), combinations of immune checkpoint inhibitors (ICIs) and VEGFR-targeted tyrosine kinase inhibitors (TKIs) are standard first-line therapies. However, the clinical benefit of these regimens in patients with favorable IMDC risk remains unclear. **Methods**: We retrospectively analyzed 147 patients with favorable-risk metastatic RCC treated with first-line systemic therapy at the Samsung Medical Center between 2019 and 2023. Treatment regimens included TKI monotherapy (*n* = 110) or ICI–TKI combinations (*n* = 37). Progression-free survival (PFS), overall survival (OS), and objective response rate (ORR) were evaluated using Kaplan–Meier and Cox regression analyses. **Results**: At a median follow-up of 46.3 months, the overall median PFS was 20.1 months (95% CI, 14.5–25.7). Median PFS was 26.2 months with ICI–TKI combinations versus 17.0 months with TKI monotherapy (HR 1.32; 95% CI, 0.82–2.12; *p* = 0.25). In multivariate analysis, TKI monotherapy (HR 14.01; *p* = 0.002) and liver metastasis (HR 9.17; *p* < 0.001) were independent predictors of shorter PFS. ORR was significantly higher with combination therapy (68% vs. 46%; *p* = 0.01). Median OS was not reached in either group, with 3-year OS rates of 89% and 84%, respectively. **Conclusions**: The findings suggest that even within the favorable-risk population, clinical heterogeneity influences treatment outcomes, emphasizing the need for individualized therapy selection and refined prognostic models.

## 1. Introduction

For patients with advanced or metastatic clear cell renal cell carcinoma (RCC), combinations of immune checkpoint inhibitors (ICIs) and vascular endothelial growth factor receptor (VEGFR)-targeted tyrosine kinase inhibitors (TKIs) have become the cornerstone of first-line systemic therapy. Multiple phase III randomized clinical trials involving ICI-TKI combinations, such as pembrolizumab plus axitinib (KEYNOTE-426) [1], pembrolizumab plus lenvatinib (CLEAR) [2], avelumab plus axitinib (JAVELIN Renal 101) [3], and nivolumab plus cabozantinib (CheckMate 9ER) [4], have demonstrated significant improvements in progression-free survival (PFS) and overall survival (OS) compared with TKI (i.e., sunitinib) monotherapy. At the same time, dual ICI therapy with nivolumab plus ipilimumab (CheckMate 214) has shown durable responses in intermediate and poor-risk patients [5]. Based on these data, treatment guidelines now recommend ICI-based combinations as standard first-line options for advanced RCC patients [6,7,8].

Risk stratification remains a key consideration in choosing the type of systemic therapy. The International Metastatic RCC Database Consortium (IMDC) scoring system is widely used to classify patients into favorable, intermediate, or poor risk based on clinical and laboratory factors such as performance status, time between diagnosis and the initiation of therapy, hemoglobin level, calcium, neutrophil, and platelet counts [9]. While the guidelines recommend ICI-TKI combinations as standard first-line therapy regardless of IMDC risk, the overall survival (OS) benefit has not been demonstrated in the IMDC favorable-risk patients [10,11,12]. Meta-analyses of pivotal trials consistently report that ICI-TKI combinations improve PFS and response rates compared with sunitinib in favorable RCC yet fail to significantly extend OS [13]. Similarly, a pooled analysis of over 3000 patients by the U.S. Food and Drug Administration reported that the OS benefit with ICI-TKI is primarily driven by intermediate and poor-risk patients [14], while the benefit remains uncertain in favorable-risk disease.

These observations suggest the biology of favorable-risk RCC may differ substantially from that of intermediate or poor-risk disease. Favorable-risk tumors tend to exhibit a more angiogenic and less inflammatory microenvironment [15], which may confer higher sensitivity to TKIs than to ICIs. Furthermore, patients with a favorable-risk RCC often present with indolent and asymptomatic disease that complicates treatment decision-making in real-world clinical practice. Factors to be considered include evidences, the extent of disease, potential toxicity, economic status, and IMDC risk scores. In this context, real-world evidence is essential to complement clinical trial data and to inform treatment decisions in routine oncology practice. In an effort to generate real-world data in Korean RCC patients, we performed a retrospective study to evaluate first-line systemic therapy for favorable-risk, advanced or metastatic, clear cell RCC.

## 2. Materials and Methods

This is a single-center, retrospective study using a prospectively collected cancer chemotherapy registry. The study protocol was reviewed and approved by the Samsung Medical Center (SMC; Seoul, Republic of Korea) institutional review board (SMC IRB no. 2021-08-054). Written informed consent was given by all patients prior to receiving systemic therapy, according to an institutional guideline. The criteria for case inclusion were as follows: (1) histologically proven clear cell carcinoma arising from kidney, (2) presence of metastatic disease, (3) no prior systemic therapy except for adjuvant treatments, (4) favorable-risk disease according to the IMDC criteria, and (5) availability of clinical data at the time of starting first-line therapy and during follow-up. Patients who were enrolled in clinical trials were excluded to ensure the choice of a specific regimen was at the discretion of the treating medical oncologists. All the data was prospectively recorded and only the survival outcome was updated at the time of analyses. 

IMDC favorable risk was described with such patients meeting all of the following criteria [9]: ≥1 year from time to diagnosis to systemic therapy, Karnofsky performance status ≥ 80%, hemoglobin ≥ lower limit of normal, and corrected calcium, neutrophil, and platelet counts ≤ upper limits of the normal range. All patients received first-line therapy involving TKI monotherapy (sunitinib or pazopanib), or ICI-TKI combinations. Dosages and schedules of each therapy were determined according to the approved guidelines. First-line therapy was continued until disease progression or lack of clinical benefit, consent withdrawal, or unacceptable toxicity. The dosage of subsequent cycles was adjusted according to the toxic effects that developed during the preceding cycle. If a patient developed disease progression, second-line therapy was recommended to all patients if their performance status was preserved. According to the guidelines and department policies, tumor assessment was performed every 3 months of therapy, by using computed tomography (CT) scans and other tests that were used to initially stage the disease. Objective response was evaluated according to the Response Evaluation Criteria for Solid Tumors (RECIST).

The endpoints of the study included OS, PFS, and response rates to first-line therapy. The starting point of OS and PFS was the first day of therapy. PFS and OS were estimated by using the Kaplan–Meier method. To examine the impact of clinical and treatment variables on the outcomes of therapy, multivariate Cox regression models were used with covariates including the initial stage at diagnosis, the presence of mixed histologic subtypes, the number of metastatic sites (one vs. ≥2), sites of metastases (liver, bone), and therapy regimens. The potential presence of interaction effects between baseline variables was tested by defining product terms for the respective variables in a regression model. All *p*-values were two-sided, with *p* < 0.05 indicating statistical significance. Analyses were performed using R for Windows 4.2.0 (https://r-project.org; accessed on 1 October 2025).

## 3. Results

### 3.1. Study Population Overview

**Figure 1:**
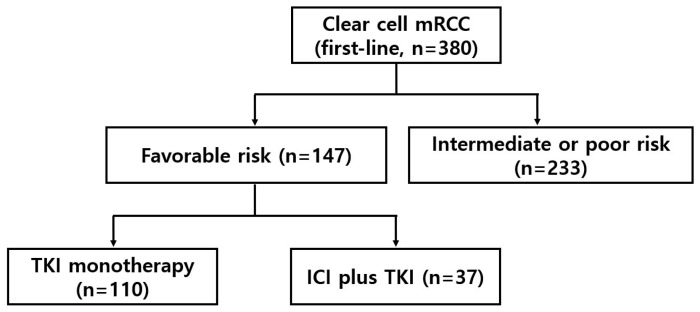
Study flow of all patients treated with first-line systemic therapy between October 2019 and December 2023. mRCC denotes metastatic renal cell carcinoma. TKI denotes tyrosine kinase inhibitor. ICI denotes immune checkpoint inhibitor.

Between October 2019 and December 2023, a total of 380 patients with advanced or metastatic RCC were consecutively treated with first-line systemic therapy at the medical oncology department of SMC. The most commonly administered first-line therapy was TKI monotherapy (sunitinib or pazopanib, *n* = 208), followed by nivolumab plus ipilimumab (*n* = 101), pembrolizumab plus axitinib or lenvatinib (*n* = 46), and nivolumab plus cabozantinib (*n* = 25). Among them, 147 patients (39%) were classified to have a favorable-risk RCC according to the IMDC criteria and were included in the present analysis (Figure 1). TKI monotherapy (either sunitinib or pazopanib) was given to 110 patients (75%), while 37 patients (25%) received ICI + TKI (nivolumab plus cabozantinib, *n* = 15; pembrolizumab plus axitinib, *n* = 14; pembrolizumab plus lenvatinib, *n* = 8). Baseline patient characteristics are summarized in Table 1. The median age at treatment initiation was 66 years, with a predominance of male patients (78%). Ninety-seven percent of patients had prior nephrectomy. The most common sites of metastases included lymph nodes and lung. Patients in the combination therapy group were slightly younger and tended to have a higher proportion of multiple metastatic sites, particularly involving the lung and liver. Liver metastasis was more frequently observed in the ICI-TKI group (16%) compared to the TKI monotherapy group (4%).

### 3.2. Oncologic Outcomes

**Figure 2:**
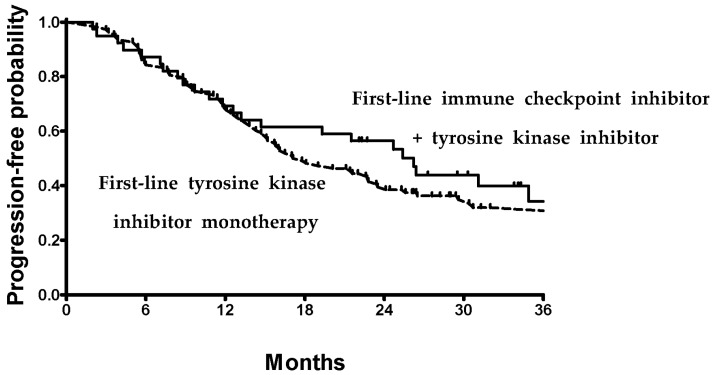
Progression-free survival. Solid lines denote patients who received first-line immune checkpoint inhibitor plus tyrosine kinase inhibitor therapy. Dotted lines denote patients who received tyrosine kinase inhibitor monotherapy.

At a median follow-up duration of 46.3 months, the overall median PFS for all favorable-risk patients was 20.1 months (95% CI, 14.5 to 25.7). Patients treated with ICI-TKI combinations achieved a numerically longer median PFS of 26.2 months compared with 17.0 months in those receiving TKI monotherapy (Figure 2). Although this difference did not reach statistical significance in unadjusted analysis (*p* = 0.25), a subsequent multivariate regression model identified type of therapy and liver metastasis as independent predictors for PFS (Table 2). Specifically, TKI monotherapy was associated with a higher risk of disease progression (HR, 14.01; *p* = 0.002), and the presence of liver metastasis conferred a markedly worse PFS (HR 14.01; *p* < 0.001). In order to ensure the robustness of the multivariate model, multicollinearity was tested using Variance Inflation Factors (VIFs). All included variables demonstrated VIFs of less than 2.5, indicating no significant multicollinearity.

The median OS was not reached in the entire cohort. At 36 months, the estimated OS rate was 89% for the ICI-TKI combination group and 84% for the TKI monotherapy group. In multivariate analysis, neither first-line therapy regimen nor baseline clinical variables demonstrated a statistically significant association with OS. Objective response was evaluable in all patients. The overall response rate was 52%, with a higher rate observed in patients receiving ICI-TKI combinations compared to TKI monotherapy (68% vs. 46%; *p* = 0.011). Complete responses were observed in 8% of patients in the combination group and 3% in the TKI monotherapy group. The disease control rate (responses plus stable disease) was 91% and 83%, respectively.

## 4. Discussion

The primary objective of this retrospective study was to evaluate the real-world outcomes of favorable-risk RCC patients treated with different first-line systemic therapy regimens. With the approval and widespread adoption of first-line ICI-TKI combinations, clinicians increasingly face the decision of whether to use these regimens in patients with favorable prognostic features. In the present analysis, the median PFS of 26.2 months observed with ICI-TKI combinations aligns well with findings of the published trials [1,2,3,4], indicating these benefits are reproducible in real-world practice. Multivariate analyses further suggested that both TKI monotherapy and the presence of liver metastasis were independently associated with inferior PFS, although interpretation of our results is limited by the retrospective nature and small sample size, highlighting that even within the favorable-risk population, patients may benefit from combination strategies. The lack of an OS difference may reflect the relatively indolent course of favorable-risk RCC and the availability of subsequent therapies after disease progression. The complete response rate and the disease control rate were higher in the combination group than in the monotherapy group. These findings indicate that while both approaches provide durable disease control, combination regimens are associated with higher response rates and deeper tumor shrinkage.

While the benefits of the ICI-TKI combinations are well established in RCC patients with intermediate and poor risk, their efficacy in favorable-risk patients is still being determined. The OS and PFS benefits of ICI-TKI over TKI monotherapy remain ambiguous in the IMDC favorable-risk patients [1,2,3,4]. Randomized trials and meta-analyses have consistently shown that ICI-TKI combinations yield higher response rates and longer PFS than TKI monotherapy, yet without a corresponding OS improvement in favorable-risk patients. This raises the need for a more refined strategy to improve patient selection criteria within the IMDC favorable-risk category to determine who truly benefits from first-line combination therapy. First, one must consider that even favorable-risk patients are heterogeneous. The IMDC risk model was initially developed and validated in the era of TKI monotherapy and may not fully capture the immunologic and molecular heterogeneity of patients treated with ICI-based regimens. Recent efforts have sought to redefine “very favorable” disease, characterized by prolonged interval from diagnosis to therapy (≥3 years), good performance status (≥90% according to Karnofsky criteria), and absence of brain, liver, or bone metastases [16]. In a real-world IMDC registry study [17], patients meeting these stringent criteria experience no OS benefit when treated with first-line ICI-TKI combination therapy. Interestingly, in the subgroup of patients with very-favorable-risk disease, there was a significantly worse 2-year OS (HR 3.19; *p* = 0.041) and objective response rate with nivolumab plus ipilimumab compared to other regimens, suggesting the importance of including TKIs in the treatment regimen for this patient subset. Evidence suggested that patients with favorable-risk disease have a more angiogenic milieu [15], which may partly explain why a TKI may be the main therapeutic option for better outcomes. It should be emphasized that favorable-risk RCC demands a completely different therapeutic approach compared to poor-risk disease, where ICIs play a more significant role than TKIs [18]. Poor-risk or even favorable-risk RCC with sarcomatoid features may feature a more inflammatory milieu [19], whereby ICI-based combination therapy would be more important than TKI monotherapy.

Another key consideration is disease kinetics and patient symptoms. Many patients with IMDC favorable-risk RCC are asymptomatic and have indolent tumor biology, raising concerns about overtreatment and quality-of-life impairment. Active surveillance without systemic therapy has emerged as a feasible strategy for select patients with asymptomatic, low-volume metastatic disease, with prospective studies showing median surveillance durations of up to 60 months before initiating systemic therapy [20,21,22]. Nevertheless, there may be patients who may prioritize complete remission of metastatic disease. In Keynote 426 and Checkmate 214 trials [5,10], ICI-based combinations achieved complete responses in approximately 11% to 12% of favorable-risk patients, suggesting a potential role for ICI-TKI combinations in those who wish for curative-intent management [23], providing they are young and have few comorbidities interfering with life expectancy.

Toxicity profiles and access to therapy are also important considerations given the balance between higher rates of immune-related adverse events associated with ICIs and the possibility of chronic toxicities with TKIs that may affect long-term tolerability. Guidelines recommend TKI monotherapy as an option if ICIs are contraindicated or unavailable [6,7]. In Korea, the absence of reimbursement for the ICI-TKI combination by the National Healthcare insurance system poses a major barrier, often leading to treatment discontinuation or selection of TKI monotherapy for financial reasons. Therefore, for symptomatic patients with IMDC favorable-risk disease requiring systemic therapy but unable to receive ICI-based combinations, TKI monotherapy remains a pragmatic and effective choice. Our findings are in line with the recently published results of the ARON-1 retrospective study [24].

Liver metastasis emerged as an adverse prognostic factor in the multivariate analysis (Table 2). The finding is consistent with the aggressive nature of liver involvement in RCC, which often reflects a unique “immune-excluded” or “immune-cold” tumor microenvironment [25]. Furthermore, the high angiogenic phenotype typically observed in favorable-risk disease may be particularly pronounced in liver metastases [15], necessitating the potent VEGFR inhibition provided by combination therapies to overcome inherent resistance. Of note, the ICI-TKI combination group in the present study had a higher baseline prevalence of liver involvement (16% vs. 4%). Despite the imbalance, which would typically favor the monotherapy group, the ICI-TKI group still achieved superior PFS.

The present study has limitations, including its retrospective, single-center design, and relatively small number of patients in the combination group. While the PFS trend strongly favors combination therapy, the exact magnitude of the HR may be influenced by the limited number of events in the favorable-risk population. In addition, the analysis of OS is premature. The lack of an OS difference may reflect the relatively indolent course of favorable-risk RCC and the availability of subsequent therapies after disease progression. However, it should be acknowledged that the present study did not follow up to assess OS for a longer period. With a median follow-up of 46.3 months and 3-year OS rates exceeding 84% in both groups, potential OS differences may not be detectable due to the inherently prolonged survival of favorable-risk patients. Nevertheless, our data provide meaningful real-world evidence that supports the potential efficacy of ICI-TKI combinations even among favorable-risk patients, while underscoring the importance of individualized approaches based on clinical and socioeconomic factors. Further research should focus on refining risk stratification incorporating molecular and immune signatures, which would guide personalized treatment selection and identify subsets of favorable-risk RCC patients who may derive a durable benefit from ICI-based combinations.

## 5. Conclusions

In summary, our real-world study showed that ICI-TKI combination therapy provided numerically longer PFS and higher objective response rates compared with TKI monotherapy in IMDC favorable-risk RCC, although OS was not different within the follow-up duration. The presence of liver metastasis emerged as an adverse prognostic factor, while therapy selection remained an independent predictor for longer PFS and disease control. These results suggest that even within the favorable-risk RCC population, clinical and biologic heterogeneity influences therapeutic outcomes.

## Figures and Tables

**Table 1:** Baseline patient characteristics.

	All Patients (*n* = 147)	TKI Monotherapy (*n* = 110)	ICI-TKI Combination (*n* = 37)	*p*-Value
Age, years				0.9377
Median (range)	66 (32 to 83)	67 (36 to 83)	61 (32 to 79)	
Gender				0.24
Male, *n* (%)	117 (79.5)	90 (81.8)	27 (72.9)	
Female, *n* (%)	30 (20.4)	20 (13.6)	10 (27.0)	
Prior Nephrectomy				>0.99
Yes, *n* (%)	143 (97.2)	107 (97.2)	36 (97.2)	
N, *n* (%)	4 (2.7)	3 (2.7)	1 (2.7)	
Stage at diagnosis				0.64
1, *n* (%)	97 (65.9)	73 (66.3)	24 (64.8)	
2, *n* (%)	22 (14.9)	18 (16.3)	4 (10.8)	
3, *n* (%)	28 (19.0)	19 (17.2)	9 (24.3)	
Mixed histologic subtypes				>0.99
Yes, *n* (%)	10 (6.8)	8 (7.2)	2 (5.4)	
No, *n* (%)	137 (93.1)	102 (92.7)	35 (94.5)	
No. of metastatic sites				<0.001
1, *n* (%)	109 (74.1)	100 (90.9)	9 (24.3)	
2 or more, *n* (%)	38 (25.8)	10 (9.0)	28 (75.6)	
Metastatic sites				
Lymph nodes, *n* (%)	38 (25.8)	25 (22.7)	13 (35.1)	0.14
Lung, *n* (%)	34 (23.1)	19 (17.2)	15 (40.5)	0.004
Liver, *n* (%)	10 (6.8)	4 (3.6)	6 (16.2)	0.01
Bone, *n* (%)	15 (10.2)	13 (11.8)	2 (5.4)	0.36
Adrenal, *n* (%)	12 (8.1)	7 (6.3)	5 (13.5)	0.18
Pancreas, *n* (%)	6 (4.0)	3 (2.7)	3 (8.1)	0.14
Brain, *n* (%)	5 (3.4)	3 (2.7)	2 (5.4)	0.59
First-line therapy	78 (53.0)	78 (70.9)		
Sunitinib, *n* (%)	32 (21.7)	32 (29.0)		
Pazopanib, *n* (%)	15 (10.2)		15 (40.5)	
Nivolumab + cabozantinib, *n* (%)				
Pembrolizumab + axitinib, *n* (%)	14 (9.5)		14 (37.8)	
Pembrolizumab + lenvatinib, *n* (%)	8 (5.4)		8 (51.6)	

Data are presented as median (range) or number (%). *p*-values compare TKI monotherapy and ICI–TKI combination groups. Categorical variables were compared using the χ^2^ test or Fisher’s exact test, as appropriate. Age differences between groups were assessed with the Mann–Whitney U test.

**Table 2:** Multivariate analyses according to baseline clinical characteristics and therapy.

	Progression-Free Survival	Overall Survival
	HR, 9.174	HR, 1.567
Liver metastasis	95% CI, 2.890 to 28.571	95% CI, 0.188 to 12.987
(yes vs. no)	*p* < 0.001	*p* = 0.678
	HR, 14.010	HR, 1.325
Therapy	95% CI, 2.619 to 74.961	95% CI, 0.112 to 16.358
TKI monotherapy vs. combinations	*p* = 0.002	*p* = 0.813

## Data Availability

The original contributions presented in this study are included in the article. Further inquiries can be directed to the corresponding author.
